# Cat scratch disease neuroretinitis: A case report

**DOI:** 10.1016/j.amsu.2021.102722

**Published:** 2021-08-15

**Authors:** Ahmed Mahjoub, Foued Bellazreg, Nadia Ben Abdesslem, Ilhem sellem, Anis Mahjoub, Syrine Ben Mrad, Mohamed Ghorbel, Amel Letaief, Mahjoub Hachmi, Krifa Fethi

**Affiliations:** a Department of Ophthalmology, Farhat Hached University Hospital, University of Medicine of Sousse, Tunisia; b Department of Infectious Diseases, Farhat Hached University Hospital, University of Medicine of Sousse, Tunisia

**Keywords:** Bartonella henslae, Cat scratch disease, Neuroretinitis, Eye infections, Doxycycline

## Abstract

**Introduction:**

Cat scratch disease (CSD) is a ubiquitous infectious disease caused by a Gram-negative intracellular bacillus, Bartonella henselae. Neuroretinitis is a classical but rare manifestation of CSD.

**Case presentation:**

A 20-year-old woman presented with a 5-day-history of reduced vision in the left eye (LE). Two weeks before eye symptoms, she complained from fever, fatigue and arthromyalgia which resolved spontaneously. In the LE, visual acuity (VA) was 7/10, fundus photography showed optic disc edema with macular exudates arranged in incomplete macular star. Serologic test for Bartonella henselae using indirect immunofluorescent assay (IFA) was highly positive (1:2560 UI/L) for immunoglobulin G (Ig G). The diagnosis of CSD associated neuroretinitis has been made and the patient was treated with doxycycline, rifampicin and oral prednisolone. Twelve months after the end of therapy, VA was 10/10, fundus photography and Macular OCT were normal.

**Discussion:**

In CSD, neuroretinitis occurs 2–3 weeks after systemic symptoms. The clinical features of CSD are not specific hence the need for bacteriological diagnosis which is based mainly on serologic testing by the detection of Ig G and Ig M by IFA or ELISA. The treatment of CSD-associated neuroretinitis is not standardized. Antibiotics active against intracellular bacteria, with or without systemic corticosteroids, should be prescribed especially in severe cases. The outcome of Bartonella henslae neuroretinitis is usually favourable.

**Conclusion:**

Despite rarely reported in Tunisia, CSD should be considered in patients with presence of typical neuroretinitis with macular star and of a history of contact with cats.

## Highlights

• Cat scratch disease is usually a self-limited lymphadenitis.
• Neuroretinitis is a rare but classic manifestation of cat scratch disease.
• Neuroretinitis occurs in 1–2% of patients with cat scratch disease.
• Diagnosis confirmation is based on serologic testing.
• The prognosis is usually good.

## 1 Introduction

Cat scratch disease (CSD) is a ubiquitous infectious disease caused by a Gram-negative intracellular bacillus, *Bartonella henselae*. It is usually transmitted by cat scratches, licks or bites, and occasionally transmitted by cat flea, *Ctenocephalides felis* [1]. Despite the most common clinical presentation of CSD is self-limited regional lymphadenitis, systemic complications such as pneumonia, meningitis, endocarditis and ocular manifestations can occur particularly in immunocompromised patients [2]. Neuroretinitis is a classical but rare manifestation of which occurs in 1–2% of patients [3]. To the best of our knowledge, only a few cases of neuroretinitis caused by *Bartonella henselae* were reported in Tunisia [4]. We report a case of CSD associated with unilateral neuroretinitis in a young immunocompetent patient.

This case report has been reported in line with the SCARE Criteria [5].

## 2 Case presentation

A 20-year-old woman with no past medical history was admitted in November 2018 to the department of ophthalmology at Farhat Hached University hospital, (Sousse, Tunisia) for a 5-day-history of reduced vision with scotoma and myodesopsia in the left eye. She had no eye pain, redness or discharge. Two weeks before eye symptoms, she complained from fever, fatigue and arthromyalgia which resolved spontaneously. Physical examination at admission was unremarkable and didn't show lymphadenopathy or skin lesion. Laboratory data showed white blood cells 10,800/mm^3^, C-reactive protein 12 mg/L and erythrocyte sedimentation rate 41 mm per hour.

**Fig. 1:**
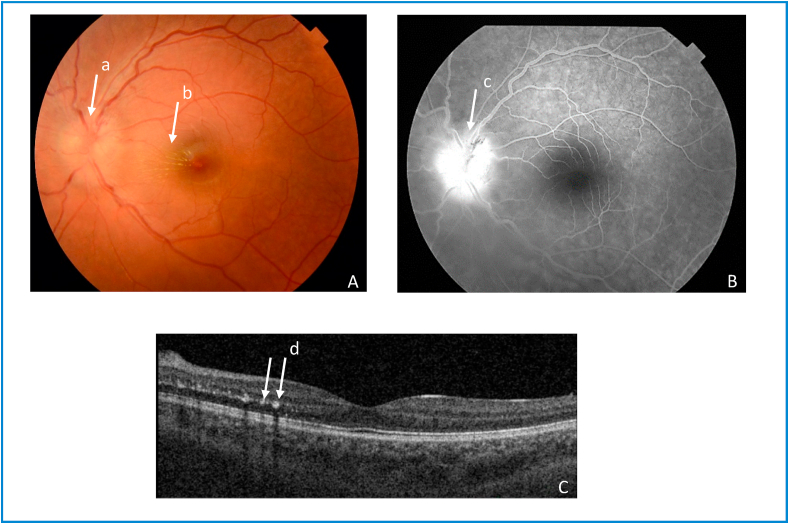
Left eye cat scratch disease neuroretinitis (at admission) A. Fundus photograph of the left eye shows optic disc edema (a) with macular exudates arranged in incomplete macular star (b). B. Late-phase FA shows optic disc hyperfluorescence (c). C. Macular OCT shows hyperreflective signals at level of the outer plexiform layer representing retinal exudates (d).

**Fig. 2:**
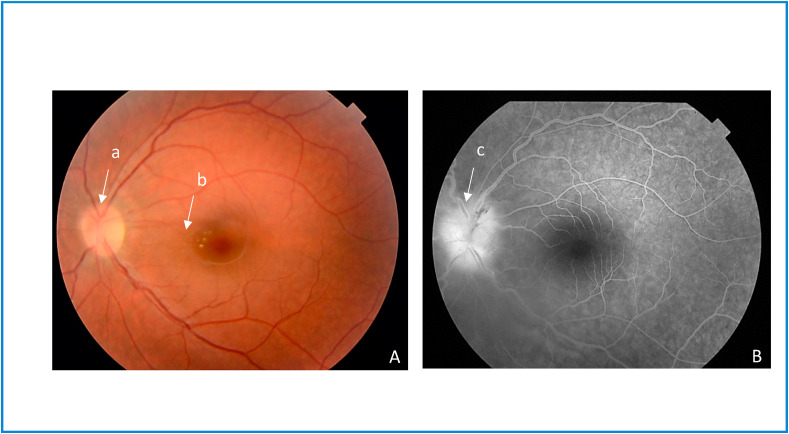
Left eye cat scratch disease neuroretinitis (at the end of antibiotic and corticosteroid therapy). A. Fundus photograph shows a decrease in optic disc edema (a) and a decrease in macular exudates (b). B. Late-phase fluorescein angiogram shows a decrease in optic disc hyperfluorescence (c).

Detailed ophthalmic examination including best corrected visual acuity (BCVA), tonometry, fundus photography, fluorescein angiography, optical coherence tomography (OCT) and visual field testing, was normal in the right eye. In the left eye, BCVA was 7/10, intraocular pressure was normal, fundus photography showed optic disc edema with macular exudates arranged in incomplete macular star, fluorescein angiography exhibited optic disc hyperfluorescence and macular OCT showed hyperreflective signals at the level of the outer plexiform layer representing retinal exudates (Fig. 1). Visual field testing showed cecocentral scotoma. Based on these findings, namely optic disc edema and macular exudates arranged in macular star, the diagnosis of neuroretinitis of the left eye was made. Chest x-ray was normal and tuberculin skin test was negative. Serologic tests for *Rickettsia*, *Brucella*, *Toxoplasma*, Cytomegalovirus (CMV), Ebstein-Barr virus (EBV), *Treponema*, *Borrelia*, and antinuclear antibodies (ANA) were all negative. Serologic test for *Bartonella henselae* using indirect immunofluorescent assay (IFA) was highly positive (1:2560 UI/L) for immunoglobulin G (Ig G), while immunoglobulin M (Ig M) were not performed. The medical history revealed that the patient had a close contact with cats and kittens and that she had multiple scratches before the onset of symptoms. The diagnosis of cat CSD associated neuroretinitis was made and the patient was treated with doxycycline 200 mg per day and rifampicin 900 mg per day, associated with oral prednisolone initially 60 mg per day, with progressive tapering for a total duration of 6 weeks. At the end of antibiotic and corticosteroid therapy, BCVA was 9/10, fundus photography showed a decrease in optic disc edema and macular exudates (Fig. 2), and fluorescein angiography exhibited a decrease in optic disc hyperfluorescence (Fig. 2).

**Fig. 3:**
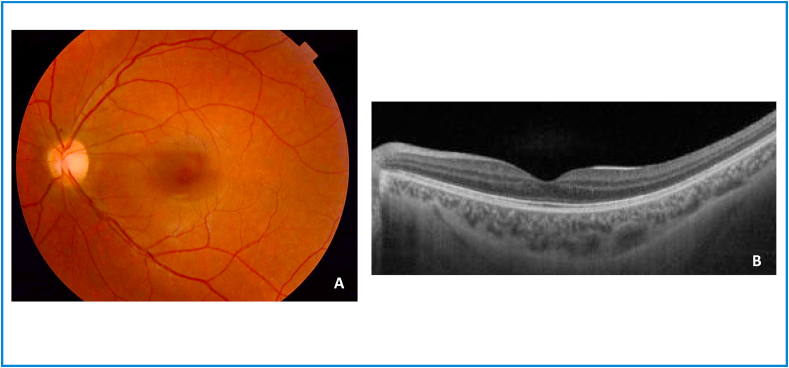
Left eye cat scratch disease neuroretinitis (12 months after the end of therapy). A. Normal fundus. B. Normal macular OCT.

Twelve months after the end of therapy, the patient was completely asymptomatic, BCVA was 10/10, fundus photography and macular OCT were normal (Fig. 3).

This case report has been reported in line with the SCARE Criteria [5].

## 3 Discussion

Neuroretinitis is an inflammation of the optic nerve and peripapillary retina characterized by optic disc edema and macular, lipid-rich, exudates arranged in a complete or incomplete star figure. It is usually unilateral and can occur in both immunocompetent and immunocompromised patients [2]. Neuroretinitis can be observed in inflammatory diseases such as systemic erythematous lupus, Behçet disease and sarcoidosis, and most frequently in infectious diseases such as rickettsioses, Lyme disease, toxoplasmosis, tuberculosis, syphilis and cat scratch disease [[6], [7], [8]]. In CSD, neuroretinitis occurs 2–3 weeks after systemic symptoms including fever and arthromyalgia. Its pathogenesis is not clear. It might be caused directly by *Bartonella henselae*, indirectly by immune response, or by both mechanisms [2]. Other ocular manifestations include Parinaud's oculoglandular syndrome, uveitis, chorioretinitis and retinal vascular occlusion. CSD should be considered in the presence of a history of contact with cats which is reported in the majority of patients while cat scratches, licks or bites are less frequently reported [[8], [9], [10]]. The clinical features of CSD are not specific hence the need for bacteriological diagnosis which is based mainly on serologic testing by the detection of Ig G and Ig M by IFA or ELISA. Ig G titers exceeding 1:256 confirm CSD while Ig M lack specificity and their clinical utility is uncertain. Culture or DNA molecular detection of *Bartonella henselae* by PCR are challenging and cannot be routinely performed [2,8,11]. In our patient who had neuroretinitis with typical macular star, the diagnosis of CSD was considered based on the presence of cat scratches and confirmed by serologic testing which showed high titers of anti-*Bartonella henselae* Ig G. Other infections and inflammatory diseases have been ruled out by laboratory data and imaging techniques. It is of note that the bacteriological diagnosis has been established with a delay of 10 days after the onset of ocular symptoms. In a French study about 12 cases of posterior segment involvement in CSD, the duration required between clinical suspicion and serologic confirmation varied from 4 to 20 days [8].

The treatment of CSD associated neuroretinitis is not standardized given the lack of randomized controlled clinical trials.

Antibiotics active against intracellular bacteria, with or without systemic corticosteroids, should be prescribed especially in severe cases and in immunocompromised patients, while in other cases the disease can recover spontaneously [2,7,11].

The most commonly used antibiotics are doxycycline alone or associated with rifampicin, and azithromycin, while second-line antibiotics include erythromycin, ciprofloxacin, trimethoprim-sulfamethoxazole and gentamicin [2,[7], [8], [9], [10], [11]]. Depending on the severity of the disease, the duration of antibiotic therapy ranges from 5 days to 9 weeks with a mean duration of 4–6 weeks [[8], [9], [10]].

Systemic corticosteroids are not recommended in routine use for patients with neuroretinitis because of uncertain benefit and significant morbidity including diabetes mellitus, hypertension and infection. Nevertheless, they should be associated with antibiotic therapy in severe cases for a mean duration of 4–6 weeks [2,[7], [8], [9], [10], [11]].

The outcome of CSD associated neuroretinitis is usually favourable namely in mild to moderate disease and in immunocompetent patients. However, ocular sequelae could persist in severe disease especially in immunocompromised patients.

In our case, we treated our patient with a combination of antibiotic therapy (doxycycline and rifampicin) and systemic corticosteroid therapy (prednisolone) for 6 weeks, the visual outcome was favourable.

## 4 Conclusion

Despite rarely reported in Tunisia, CSD should be considered in patients with ocular manifestations, especially in the presence of typical neuroretinitis with macular star and of a history of contact with cats. The diagnosis is confirmed by serologic testing. The treatment, indicated especially in severe cases and in immunocompromised patients, is based on antibiotic therapy and systemic corticosteroids. The outcome is often favourable.

## Sources of funding

None.

## Consent

Written informed consent was obtained from the patient for publication of this case report and accompanying images. A copy of the written consent is available for review by the Editor-in-Chief of this journal on request.

## Guarantor

Ilhem Sellem MD.

### Ethical approval

Research studies involving patients require ethical approval. Please state whether approval has been given, name the relevant ethics committee and the state the reference number for their judgement.

All procedures performed in studies involving human participants were in accordance with the ethical standards of the institutional and/or national research committee and with the 1964 Helsinki declaration and its later amendments or comparable ethical standards.

## Declaration of competing interest

None.
